# Fatty liver disease and hypertransaminasemia hiding the association of clinically silent Duchenne muscular dystrophy and hereditary fructose intolerance

**DOI:** 10.1186/1824-7288-38-64

**Published:** 2012-10-31

**Authors:** Giulia Paolella, Pasquale Pisano, Raffaele Albano, Lucio Cannaviello, Carolina Mauro, Gabriella Esposito, Pietro Vajro

**Affiliations:** 1 Chair of Pediatrics, School of Medicine, University of Salerno, Salerno, Italy; 2 Pediatrics, University Hospital “San Giovanni di Dio e Ruggi d’Aragona”, Salerno, Italy; 3 Physical and Rehabilitative Medicine, University Hospital “San Giovanni di Dio e Ruggi d’Aragona”, Salerno, Italy; 4 Department of Biochemistry and Medical Biotechnologies, University of Naples Federico II, Naples, Italy; 5 Chair of Pediatrics, School of Medicine, University of Salerno, Via S. Allende, 84080 Baronissi, Salerno, Italy

**Keywords:** Hypertransaminasemia, Fatty liver, Hereditary fructose intolerance, Muscular dystrophies

## Abstract

We report a case with the association of well self-compensated hereditary fructose intolerance and still poorly symptomatic Duchenne type muscular dystrophy. This case illustrates the problems of a correct diagnosis in sub-clinical patients presenting with “cryptogenic” hypertransaminasemia.

## Introduction

The epidemics of obesity at all ages is still relentless. The cause of an incidental and persistent finding of fatty liver and/or cryptogenic isolated mild hypertransaminasemia (HTS) in a child may therefore tend to be more and more undervalued and wrongly included within the spectrum of "non alcoholic fatty liver disease" (NAFLD) [1].

Differential diagnosis with other more rare, and sometimes poorly known metabolic/genetic, conditions [2-6] in fact is imperative to avoid these patients not to profit of specific, possibly lifesaving, treatments [7]. An exceedingly high index of suspicion is therefore required, also because multiple causes sharing identical pathological findings exist [8]. Here we present a case of cryptic and well self-compensated hereditary fructose intolerance whose identification was triggered by the accidental finding of muscular hypertransaminasemia due to a still poorly manifest Duchenne type muscular dystrophy.

## Case report

CS, a 3-year and 4-month old boy, was admitted to our teaching hospital for HTS. He was the full term, second-born of non-consanguineous parents. He pronounced his first words after the first year, and took autonomous first steps at 24 months. Sugar, sweets and fruit refusal was reported since early life.

The first occasional discovery of HTS at age 2 years (Alanine aminotransferase x 5 upper normal values (unv), Aspartate aminotransferase x 6 unv) was confirmed at 3 subsequent outpatient checks. At entry physical examination showed a well-cooperating boy, with a hypo-mimic face. Height, weight and head circumference were normal for age and gender. Abdomen was expanded and tense. Liver and spleen were palpable at 5 and 1 centimeters under the costal ridges, respectively. Liver consistency was reduced. Mild bilateral gastrocnemius pseudohypertrophy was noticed. Gower sign was positive with discrete weakness of legs antagonists, making impossible to climb the stairs without the help of the upper limbs, and use of supports for both the transition from sitting position to standing, and for walking (wide base, accentuation of the lumbar lordosis).

Bilirubin, Gamma-glutamyl transpeptidase, Albumin and Protein serum electrophoresis, Prothrombin Time, Partial Thromboplastin Time, Fibrinogen, Blood gases, Lactic acid, Ammonium, Cholesterol, Triglycerides, Total Immunoglobulins, Viral hepatitis markers (HBsAg, anti HBc Ag, anti-HCV, anti-HAV, anti-HAV IgM), TOxoplasmosis, Rubella, Cytomegalovirus, and Herpes simplex and Mononucleosis serology, Vidal Wright, anti-nuclear antibodies, anti-mitochondrial antibody, anti-smooth muscle antibodies, anti-liver-kidney microsomes-1 antibodies, antiparietal cell antibodies, anti-endomysial antibodies IgA, anti transaglutaminasi IgA, TSH, FT3, FT4, serum and urinary amino acids and acylcarnitines were all negative or within normal limits for age. In addition to aminotransferase values, also creatine kinase (CK), lactic dehydrogenase, and myoglobin values resulted pathological (Table 1).

**Table 1:** Abnormal biochemical laboratory investigation at entry

**Test**	**Value**	**Normal values**
Aspartate aminotransferase	245 IU/L	≤ 41 U/I
Alanine aminotransferase	224 IU/L	≤ 45 U/I
Lactic acid dehydrogenase	2,268 IU/L	≤ 882 U/I
Creatine kinase	18,369 IU/L	≤ 190 U/I
Myoglobin	1,524.9 ng/ml	0.6-6.3 ng/ml

**Figure 1:**
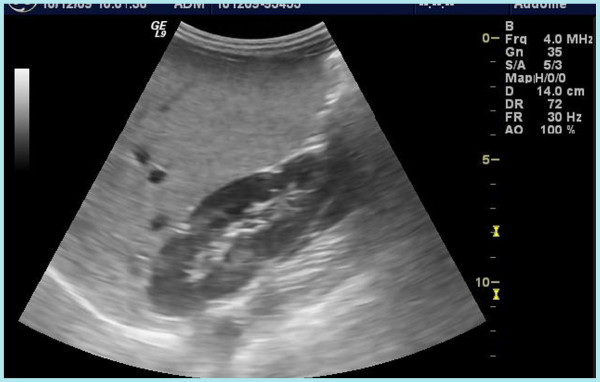
Abdominal ultrasound of CS with evidence of bright liver suggestive of severe hepatic steatosis.

Abdominal ultrasounds confirmed hepatomegaly (diameter of the longitudinal middle lobe = 14 centimeters), with diffuse steatosis. Intra and extrahepatic biliary tract were not dilated (Figure 1). The spleen volume was modestly increased (longitudinal and transverse diameter of 9.5 and 5 centimeters, respectively) with a regular echo structure.

Echocardiogram and electrocardiography did not show any gross abnormalities.

The molecular analysis for dystrophinopathies showed the patient be hemizygous for the macrodeletion comprising exons 49–50 of the dystrophin gene, compatible with Duchenne/Becker Muscular Dystrophy. His mother was heterozygous for the same macro-deletion.

Due to the existence of the association fatty liver and sweet foods dislike/aversion, the molecular analysis for the hereditary fructose intolerance (HFI), was also performed. The patient eventually resulted heterozygous for mutations pA150P and pY204X (c.612T> A), respectively localized in exons 5 of the gene ALDOB. In order to confirm that the 2 mutations were present in trans (on two different alleles) the molecular analysis was performed also in the two parents. This showed a compatible genotype with heterozygous carrier status.

At hospital discharge, due to absence of evident muscle retraction (prevalence of hyposthenia), no need for immediate physiatric treatments was agreed. A fructose/sucrose/sorbitol free diet along with a list of allowed/forbidden drugs was prescribed.

## Discussion

Our case describes for the first time the association of hereditary fructose intolerance and Duchenne muscular dystrophy, and provides the opportunity of discussing the difficulties in the evaluation of the finding *elevated transaminase* levels in a still poorly symptomatic/asymptomatic patient. This may represent a significant diagnostic challenge for physicians, given the exceedingly wide range of possible infectious, autoimmune, metabolic, systemic, gastrointestinal causes of liver disease, and also of extrahepatic origin hypertransaminasemias [8]. A detailed evaluation of the initial history, and a clinically oriented examination are crucial to assess the severity, and to determine the likely etiology/etiologies of hypertransaminasemia [8].

If hyperCKemia accompanies hypertransaminasemia it is mandatory to exclude muscular diseases, which are often clinically asymptomatic during the first 5–6 years of life and are frequently recognized only after a detailed and oriented neurologic examination. Duchenne muscular dystrophy (Phenotype MIM number 310200) is caused by mutation in the gene encoding dystrophin (DMD; 300377) with location on Xp21.2-p21.1 [9]. In a 12-year prospective study in the Campania region of southern Italy, Nigro et al. [10] found an incidence of DMD of 21.7 per 100,000 male live births. In addition to the classical muscular dystrophies (dystrophinopathies), myocyte injury and necrosis induced by drugs or toxins, increased exercise, and some mitochondrial, endocrine and metabolic (e.g. storage diseases) myopathies, and gluten enteropathy are however possible additional causes of hyperCKemia and hypertransaminasemia, needing accurate exclusion according to age and clinical scenario [2,11,12]. The association between muscular dystrophy and obesity related NAFLD has been recently reported as well, and it emerges the need for a specific handling to avoid further muscular damage consequent to excessive caloric restriction or exercise [12]. The associated condition presented by our patient, HFI (gene locus ALDOB, location 9q31.1, Phenotype Fructose intolerance, MIM number 229600) [13] has an incidence of 1:20,000. In addition to occur with a possible typical presentation pattern of early-onset cholestasis during weaning, it may also present later on in patients who spontaneously follow a low fructose diet as a result of innate fructose dislike. In these cases, medical observation may be consequent to an incidental finding of hypertransaminasemia, and/or hepatomegaly and/or bright liver at ultrasound observation. The correct feeding history is crucial for the diagnosis, which may be confirmed by molecular analysis of the gene mutations as in our case. The association of these two rare diseases has never been previously reported: the different chromosomes involved and the parental carrier state suggest that it is a fortuitous event.

In conclusion our case emphasizes that when hypertransaminasemia and/or fatty liver are diagnosed one should nonetheless be alert to the possibility of overlooking a series of other, possibly treatable, coincidental causes of liver damage such as celiac disease [14,15], Wilson disease [16], autoimmune hepatitis [17], several genetic rarer diseases [18-20] or even muscular diseases. Prompt recognition of some of these associated conditions may be crucial to avoid invasive tests [21,22], and immediately start appropriate therapies [23,24] that might dramatically change the natural history of the associated hidden cause of liver disease.

## Consent

Written informed consent was obtained from the patient's family for publication of this Case report and any accompanying images. A copy of the written consent is available for review by the Editor-in-Chief of this journal.

## Abbreviations

CK: Creatine kinase; FT3: Free Triiodothyronine; FT4: Free Thyroxine; HAV: Hepatitis A virus; HBcAg: Hepatitis B core Antigen; HBsAg: Hepatitis B surface Antigen; HCV: Hepatitis C virus; HFI: Hereditary fructose intolerance; HTS: Hypertransaminasemia; NAFLD: Non alcoholic fatty liver disease; TSH: Thyroid-stimulating hormone; UNV: Upper normal values.

## Competing interests

The authors declare that they have no competing interests.

## Authors’ contributions

PV and GP drafted the manuscript; PP, RA, CM and PV followed the patient during hospitalization. LC was the consultant physiatrist. GE carried out the molecular and genetic studies. All authors read and approved the final manuscript.
